# A Strawberry *KNOX* Gene Regulates Leaf, Flower and Meristem Architecture

**DOI:** 10.1371/journal.pone.0024752

**Published:** 2011-09-20

**Authors:** Mithu Chatterjee, Claudia L. Bermudez-Lozano, Maureen A. Clancy, Thomas M. Davis, Kevin M. Folta

**Affiliations:** 1 Graduate Program in Plant Molecular and Cellular Biology, Horticultural Sciences Department, University of Florida, Gainesville, Florida, United States of America; 2 Department of Biological Sciences, University of New Hampshire, Durham, New Hampshire, United States of America; University of Oxford, United Kingdom

## Abstract

The *KNOTTED-LIKE HOMEODOMAIN* (*KNOX*) genes play a central role in maintenance of the shoot apical meristem. They also contribute to the morphology of simple and compound leaves. In this report we characterize the *FaKNOX1* gene from strawberry (*Fragaria spp.*) and demonstrate its function in trasgenic plants. The *FaKNOX1* cDNA was isolated from a cultivated strawberry (*F.×ananassa*) flower EST library. The sequence is most similar to Class I *KNOX* genes, and was mapped to linkage group VI of the diploid strawberry genome. Unlike most *KNOX* genes studied, steady-state transcript levels were highest in flowers and fruits. Transcripts were also detected in emerging leaf primordia and the apical dome. Transgenic strawberry plants suppressing or overexpressing *FaKNOX1* exhibited conspicuous changes in plant form. The *FaKNOX1* RNAi plants presented a dwarfed phenotype with deeply serrated leaflets and exaggerated petiolules. They also exhibited a high level of cellular disorganization of the shoot apical meristem and leaves. Overexpression of *FaKNOX1* caused dwarfed stature with wrinkled leaves. These gain- and loss-of-function assays in strawberry functionally demonstrate the contributions of a KNOX domain protein in a rosaceous species.

## Introduction

Over the past decade elegant studies in *Arabidopsis thaliana* have unveiled the intricate programs that govern leaf development and morphology. These findings have been translated to tomato and other species, where new interactions with the environment and expansion of the *Arabidopsis* model have been described [1], [2]. In general, leaves show shape and size variations that reflect their adaptation to various environmental conditions and ecological contexts. Leaves can be generally classified as either simple or compound based on their arrangement and complexity. A simple leaf has a single blade at a node and can be deeply lobed or dissected, whereas, a compound leaf consists of two or more separate blades (leaflets) borne from a common rachis [3], [4]. Various hypotheses have been presented to describe the development of compound leaves [3], [5], [6], [7]. One view suggests compound leaves as intermediate structures that share similarity with both shoots and leaves. Thus, the leaflets of compound leaves are equivalent to simple leaves [8]. The other view proposes that the entire compound leaf is a simple leaf with a highly-divided leaf blade [9].

Some of the first documented scientific inquiry exploring the variation between the simple and compound leaf came from diploid strawberry (*F. vesca*). A French teenager named Antoine Duchesne examined strawberries in the royal botanical gardens in Versailles. In 1763 he identified a variant possessing single-bladed leaves that produced plants presenting only single-bladed leaves when propagated by seeds or runners [10]. Later, in 1914, C. W. Richardson would cross this same variant line against trifoliate strawberry and demonstrate that the characteristic leaf phenotype was likely controlled by a recessive mutation [10]. Today strawberry is a potentially useful system to expand the understanding of compound leaf development. As the seedling grows, its first leaves emerge as single blades, followed by a transition to the familiar mature trifoliate pattern once several simple leaves have emerged [11]. Some *Fragaria* species (e.g. *F. pentaphylla*) possess three major leaflets and two reduced ones for a total of five. When flowering, a single-bladed leaf known as a flag leaf often emerges to accompany a floral pedicle. The changes in leaf complexity observed during early development suggest that strawberry maintains complex networks of regulators that may expand the understanding of leaf development in plants.

The *KNOTTED-LIKE HOMEODOMAIN* (*KNOX*) genes, known for their contribution to maintenance of the shoot apical meristem (SAM), have also been shown to function in leaf initiation and regulation of leaf form [1], [12], [13], [14], [15]. In simple-leafed plant species such as *Arabidopsis* and maize, Class I *KNOX* genes are exclusively expressed in the SAM and are down-regulated at the early stages of leaf development. Mutations of *Arabidopsis* Class I *KNOX* gene, *SHOOTMERISTEMLESS* (*STM*) affect embryonic shoot meristem formation [12], [16]. Overexpression of these genes generates pronounced leaf lobing and ectopic meristem formation [17], [18], [19], [20]. Contrary to the situation in simple leaves, the Class I *KNOX* genes are expressed in the compound leaves of species like tomato (*Solanum lycopersicum*), bittercress (*Cardamine hirsuta*) and pepperweed (*Lepidium perfoliatum*) [2], [21], [22], [23], [24]. Overexpression of Class I *KNOX* genes in tomato results in excessive proliferation of leaflets [21], [22]. Studies on simple and complex-leaved species of *Lepidium* indicate that the final leaf morphology does not always correlate with early Class I *KNOX* expression patterns [1], [2]. For example, *Lepidium oleraceum* generates simple leaves but the Class I *KNOX* expression is not down-regulated at the early leaf primordium stage. Detailed analyses revealed that leaves show a typical compound leaf development pattern during early development. Comparative investigation on Class I *KNOX* genes from tomato and *Cardamine hirsuta* suggests a highly balanced spatial and temporal action as well as differential activity generated by upstream gene regulatory sequences regulate the leaf development [23], [25]. Thus, changes in the expression of Class I *KNOX* accompany the progression of development in the leaf.

The role of Class I *KNOX* genes has been studied in species related to strawberry. The *KNOPE1* and *KNOPE3* genes have been identified as pivotal regulators of specific physiological processes in peach (*Prunus persica*). *KNOPE1* transcripts are elevated in leaves infected with *Taphrina deformans*, leading to leaf curl [26]. Expression patterns of *KNOPE3* suggest a role in carbohydrate translocation in addition to organ development [27]. Transgenic plum (*Prunus domestica*) plants overexpressing apple *MdKN1* showed reduction in plant height and generated small malformed curly leaves [28].

In the present study, a *KNOX* gene (*FaKNOX1*) was isolated from a cultivated strawberry (*F. × ananassa*) flower EST library. The *Fa*KNOX1 protein shows similarity to Class I KNOTTED-LIKE HOMEODOMAIN proteins, with 60% identity to maize LIGULELESS3. *FaKNOX1* expression is mostly observed in the fruits and flowers, as well as in runner tips, single-bladed leaves and developing trifoliate leaves. Transgenic strawberry plants demonstrating the effects of loss and overexpression of this gene, and overexpression phenotypes in *Arabidopsis* are presented. This work demonstrates the *in planta* effect of a *KNOX* gene in strawberry leaf development and compares the findings to those observed in other species.

## Results

### Structural organization of *FaKNOX1*

**Figure 1:**
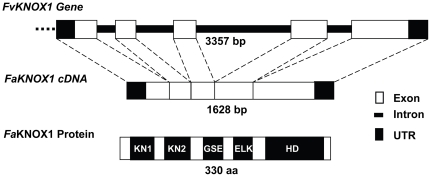
Structural organization of *FaKNOX1* gene. The schematic diagram represents the exon/intron boundaries of *FaKNOX1* cDNA and its corresponding genomic sequence. The *FaKNOX1* cDNA encodes a protein containing four domains: MEINOX domain, the GSE domain, the ELK domain and the homeodomain.

**Figure 2:**
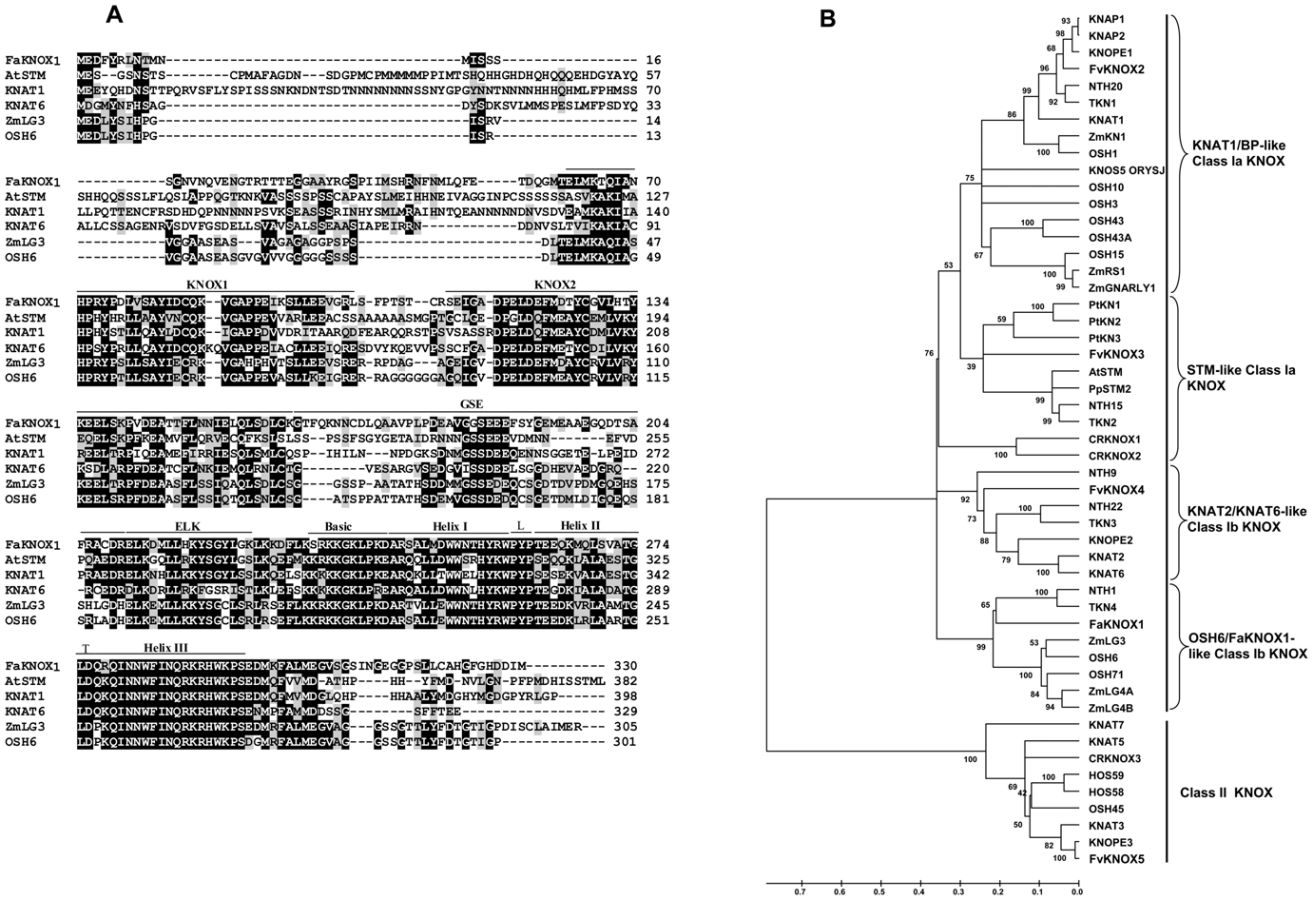
Analyses of Class I *KNOX* genes. (A) Alignment of *Fa*KNOX1 amino acid sequence along with five representatives of Class I KNOX transcription factors. Black boxes indicate identical residues and the grey boxes indicate similar residues. The lines above the sequence mark the KNOX1, KNOX2, GSE and ELK domains and the homeodomain. As characteristic of TALE superclass transcription factors the homeodomain is represented by a three-amino-acid loop extension present between the first and second α-helices of the homeodomain. The letter ‘L’ and ‘T’ represent loop and turn, respectively. (B) The phylogenetic analysis of *KNOX* genes. Class II KNOX proteins were used as outgroup. Bootstrap values based on 1000 replicates are shown next to the branching points.

The full-length *FaKNOX1* cDNA (accession number GQ465832) was isolated from a flower cDNA library prepared from *F. × ananassa* cv. Strawberry Festival. The associated genomic sequence (accession number GU339211) was PCR-amplified from the diploid *F. vesca* genome and showed 98.7% identity to the *FaKNOX1* cDNA sequence. Comparison of the cDNA with genomic sequence revealed that the *FaKNOX1* gene contains four introns and encodes a protein of 330 aa (Figure 1). *Fa*KNOX1 shares 62% identity with TKN4 and NTH1, 52% with *Arabidopsis* KNAT2 and KNAT6 [29] and 60% with closely related monocot genes, ZmLG3 and OSH6 [30], [31]. *Fa*KNOX1 maintained the same domain structure as other eudicot (*At*STM, KNAT1 and KNAT6) [12], [17], [29] and monocot species (OSH6, ZmLG3) [30], [31]. *Fa*KNOX1 possesses all three highly conserved domains typical to the KNOX proteins: the MEINOX domain that is subdivided into KNOX1 and KNOX2; the ELK domain and the homeodomain (Figure 2A). *Fa*KNOX1 also contained a GSE domain between the MEINOX and ELK domains, similar to previous reports [32]. Phylogenetic clustering of KNOX protein sequences from diverse species positioned *Fa*KNOX1 with Class I KNOX proteins, within a subgroup containing LIGULELESS and OSH6 (Figure 2B).

### Linkage Mapping of *FaKNOX1*

Diploid strawberry possesses a well-covered linkage map [33]. Recent definition of a bin mapping set provides a means to rapidly assign a linkage position to a given gene [34]. The *FaKNOX1* gene was mapped on the basis of DNA sequence differences between mapping parents Fn601 and Fv815. Comparison to the bin mapping progeny set indicated that *FaKNOX1* be assigned to Linkage Group VI, Bin 14.

### Expression pattern of *FaKNOX1*

#### *FaKNOX1* transcripts are most abundant in the flower and fruit

**Figure 3:**
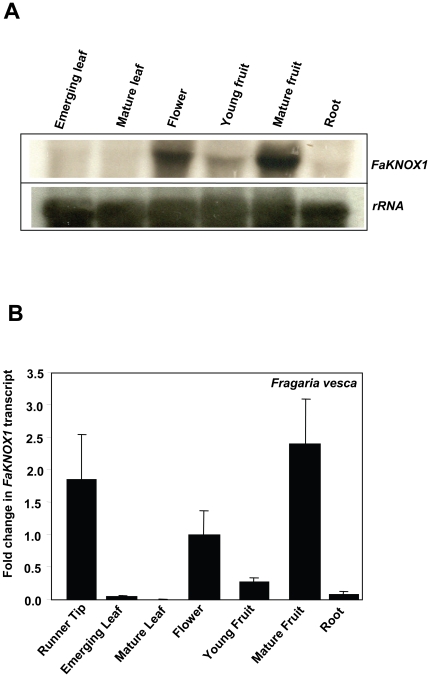
Steady-state *FaKNOX1* transcript accumulation patterns in various organs. (A) Results obtained by RNA-gel-blot analysis and hybridization. (B) A corresponding comparative quantitative real-time PCR analysis. The flower sample was used as reference tissue and EF1a was used as reference gene. Error bars represent standard error of the mean derived from three replicates.

RNA-gel-blot analysis and real-time PCR were employed to assess the tissue- specific accumulation pattern of *FaKNOX1* mRNA. *FaKNOX1* transcripts were more abundant in flower and fruit tissues than in leaves and roots (Figure 3A). Compared to the flower reference sample, relative real-time PCR results showed two- to three-fold increased *FaKNOX1* transcript abundance in runner tips (containing vegetative shoot apex) and mature fruits, while *FaKNOX1* transcript accumulation was considerably less in emerging and mature leaves (Figure 3B).

#### *FaKNOX1* transcripts are present in simple and compounds leaves

**Figure 4:**
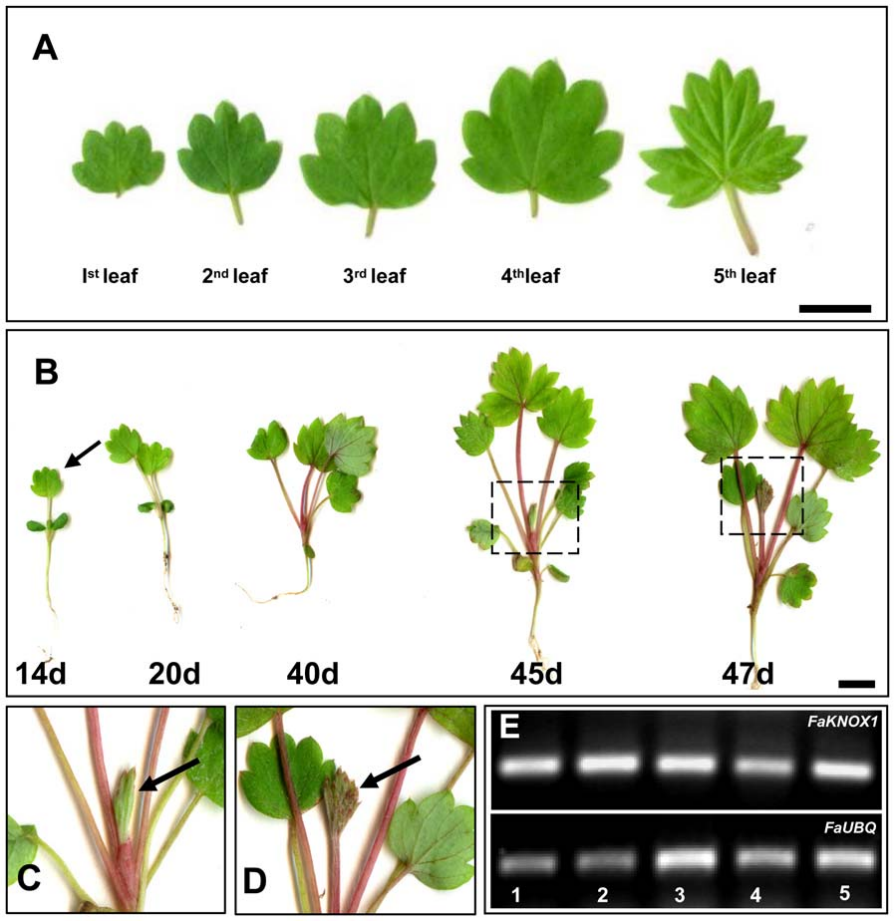
Leaf development pattern of strawberry seedlings. (A) The first four to five leaves of strawberry seedlings are simple, and then follow the familiar trifoliate leaves. (B) Development of strawberry (*F. vesca*) seedling showing the progression of leaf morphogeneisis from simple leaf to compound trifoliate leaf form. (C and D) Represent an enlarged view of developing stages of emerging trifoliate leaves (dotted regions on panel B). The arrow indicates the specific portion of leaf used for semi-quantitative RT-PCR analysis. (E) A comparative semi-quantitative RT-PCR showing that *FaKNOX1* transcript was readily detectable in leaf samples harvested from 14 d to 47–50-d-old strawberry plants. Lanes 1–3 represent expression of *FaKNOX1* in simple leaves of 14-d to 40-d-old seedlings and lanes 4–5 represent expression of *FaKNOX1* in emerging trifoliate leaves of 45- and 47-d-old seedlings. *FaUBQ* was used as an internal control gene. Bars = 5 mm.

To understand the role, if any, of *FaKNOX1* in the developmental or morphological changes in strawberry leaves, it was important to assess transcript abundance in different stages of leaf development. In strawberry the first four to five leaves are always simple, then the trifoliate pattern is followed [11]. The progression of strawberry leaf development from single-leaflet to trifoliate is depicted in Figure 4. In developing seedlings of *F. vesca* Hawaii-4, RT-PCR analyses revealed that *FaKNOX1* transcripts were readily detected at approximately the same level from the early single-leaf form to an actively expanding compound leaf (Figure 4E). Transcript levels were low in these seedling leaf tissues as the *FaKNOX1* amplicons were detected only after 38 cycles.

**Figure 5:**
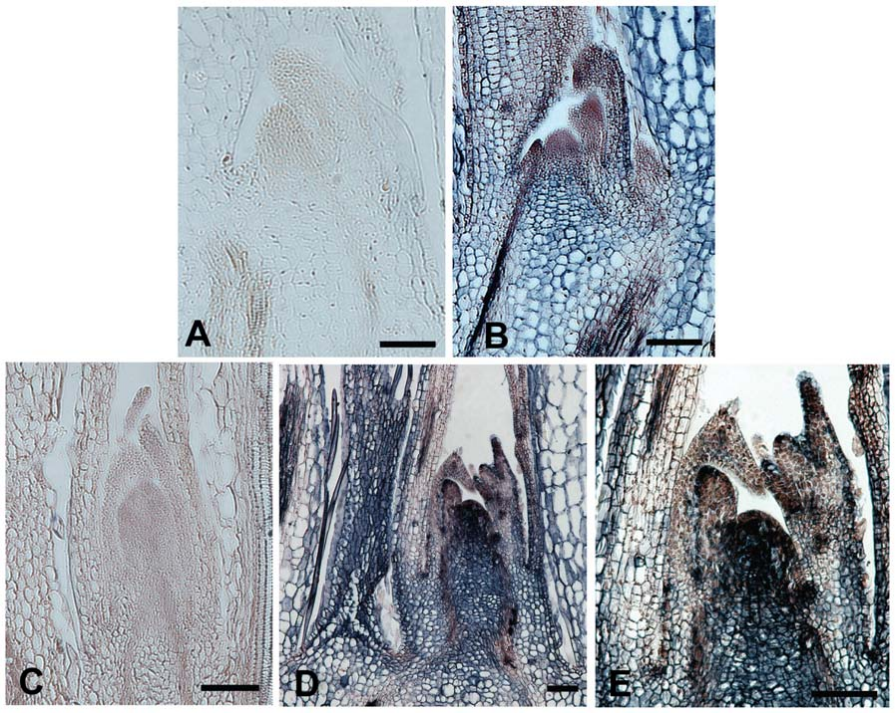
*In situ* hybridization of *FaKNOX1* in the vegetative meristem of strawberry seedlings. The presence of *FaKNOX1 is* indicated by blue/black staining. Longitudinal section through the SAM of 2-leaf-stage seedling hybridized to a sense *FaKNOX1* RNA probe (A) or antisense *FaKNOX1* RNA probe (B). Longitudinal section through the SAM of 45-d leaf-stage seedling with emerging trifoliate leaf hybridized with a sense *FaKNOX1* RNA probe (C) or an antisense *FaKNOX1* probe (D and E). Bars = 1000 µm.

To determine if *FaKNOX1* is expressed in discrete regions of the SAM and leaf primordia during leaf development, *in situ* hybridization was performed. Two leaf developmental stages were tested: 20-day-old seedlings (with simple leaves) and 45-d-old seedlings (with trifoliate leaf emerging). At the two-leaf-stage, the *FaKNOX1* transcript was detected at higher levels in the rib meristem region of SAM and lateral developing leaf (Figure 5A, B). As leaf development progressed to the trifoliate stage, the *FaKNOX1* transcript became more abundant in the dome of the SAM and at the base and outer layers of the developing leaf. Low-level expression of *FaKNOX1* was also observed within leaf primordia (Figure 5C, D, E).

### Analysis of *FaKNOX1* RNAi transgenic plants

To study the function of *FaKNOX1 in planta*, loss- and gain-of-function transgenic plants were generated. For RNAi suppression constructs two copies of the full-length *FaKNOX1* cDNA were placed under control of the cauliflower mosaic virus 35S (CaMV 35S) promoter in a head-to-head configuration. The RNAi construct was introduced into the diploid strawberry *F. vesca* Hawaii-4 using slight modifications of published protocols [35]. More than twenty independent T0 transgenic lines were analyzed. RT-PCR analysis from flower or runner tip tissues of RNAi plants showed no detectable transcript (several lines depicted in Figure S1). To verify the specificity of the RNAi construct, levels of other *KNOX-*encoding transcripts were tested. A computational analysis was performed to identify other *KNOX*-like sequences in the strawberry genome (www.strawberrygenome.org/www.rosaceae.org). Based on the BLASTn search, four related genes (noted as *FvKNOX2 - FvKNOX5*) were identified. However, these genes shared limited homology at a stretch of 80-30 nucleotides ranging from 86–96% identity (Figure 2B). Tissue-specific expression analysis indicates that transcripts for all of these genes were present in runner tips. *FvKNOX2* and *FvKNOX3* were abundantly expressed in mature fruits whereas barely detectable in leaf tissue. *FvKNOX5*, a member of Class II KNOX genes was present to a detectable label in all the tissue examined (Figure S4). To test if perturbation of *FaKNOX1* expression affected other *KNOX* genes, accumulation of *FvKNOX2 - FvKNOX5* transcripts was monitored using relative quantitative real-time PCR analysis with mRNA isolated from runner tips of four *FaKNOX1* RNAi independent lines. The data show that expression of other *KNOX* family members is not increased significantly and that the slight repeatable differences observed are the partial suppression of the *FvKNOX2* paralog transcripts (Figure S2). Thus the data indicate that the transgenic phenotypes observed may be mostly, if not completely, attributed to the suppression of the *FaKNOX1* transcript with minimal changes in other transcript levels.

**Figure 6:**
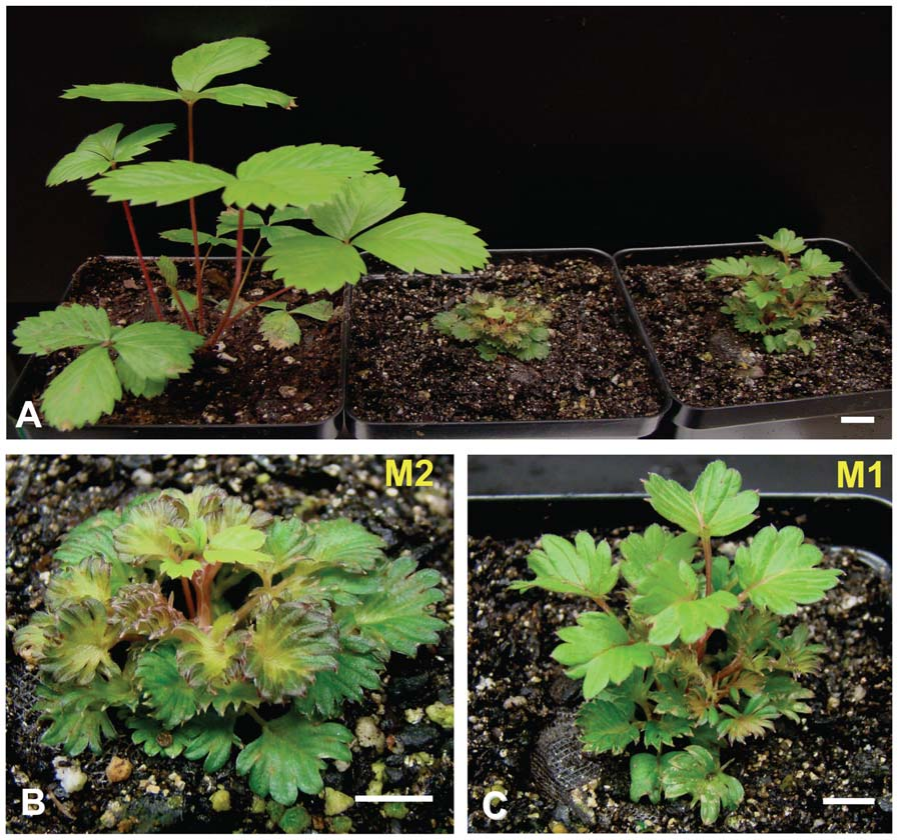
*FaKNOX1* RNAi mutants. (A) Phenotypic comparison of wild-type *F. vesca* plants with two representatives of the severe (M2) and conspicuous (M1) categories of *FaKNOX1* RNAi transgenic lines. Representative of (B) M2 *FaKNOX1* RNAi plants and (C) M1 *FaKNOX1* RNAi plants. Bars = 1 cm.

**Figure 7:**
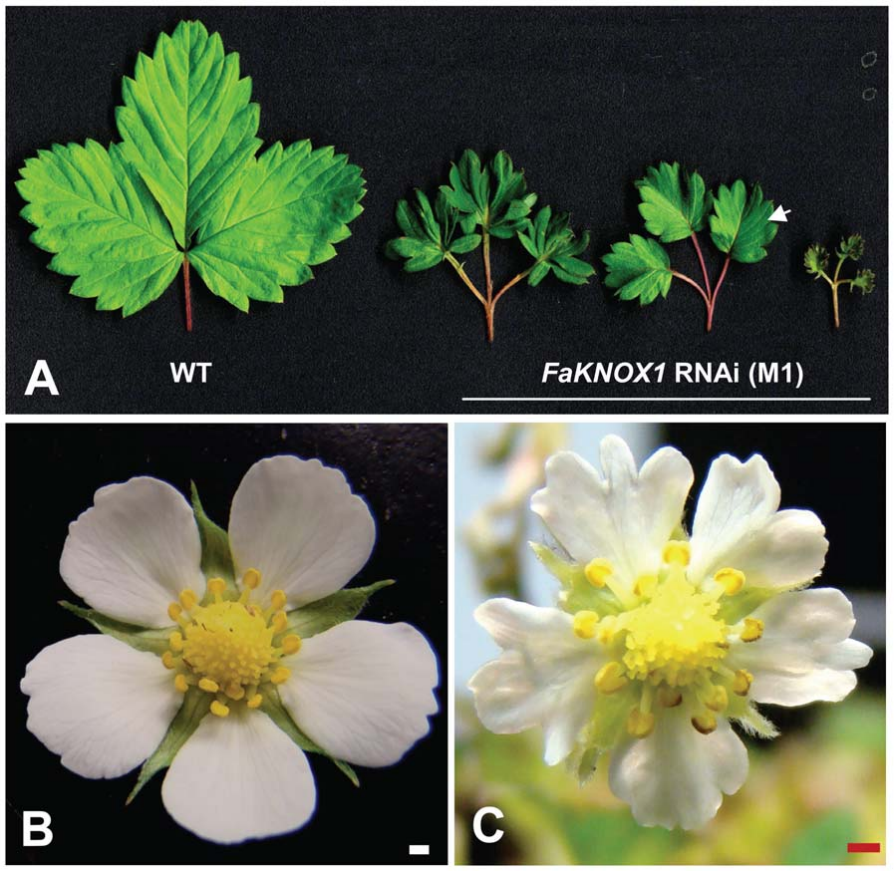
Leaf and flower morphology of a *FaKNOX1* RNAi line. (A) A wild-type leaf compared to different leaf forms of *FaKNOX1* RNAi plants. The arrow indicates the leaf portion used for cross sections in Figure 7B. (B) Wild-type strawberry flower. (C) *FaKNOX1* RNAi flower. Bars = 1 mm.

The effect of loss-of-function was evident early in plant regeneration. Whereas organogenesis from *F. vesca* callus first produces simple leaves, mostly the *FaKNOX1* RNAi callus generated trifoliate leaves directly from the callus (not shown). The resulting transgenic plants were grouped into two classes based on severity of phenotype. These classes were designated as M1 (conspicuous effect) and M2 (severe effect) (Figure 6A). The RNAi plants from class M1 (Figure 6C) presented a dwarf stature with three types of leaf form as shown in Figure 7A. The M2 transgenic plants (Figure 6B) were extremely small in size with compact rosette-like stature and produced one form of thick, highly invaginated parsley-like leaves. This leaf form was common among both M1 and M2 plants. Compared to wild-type, the leaflets of RNAi plants exhibited elongated petiolules (Figure 7A) and plants occasionally produced small flowers with lobed petals (Figure 7B, 7C). While flowers were complete, they failed to produce fruits. These plants generated runners that gave rise to daughter plants exhibiting pale colored, narrow and elongated leaves. The developing clonal daughter plants emerging from the runner buds also presented leaves from two or three distinct phenotypic classes based on size, lobing and petiolule length.

**Figure 8:**
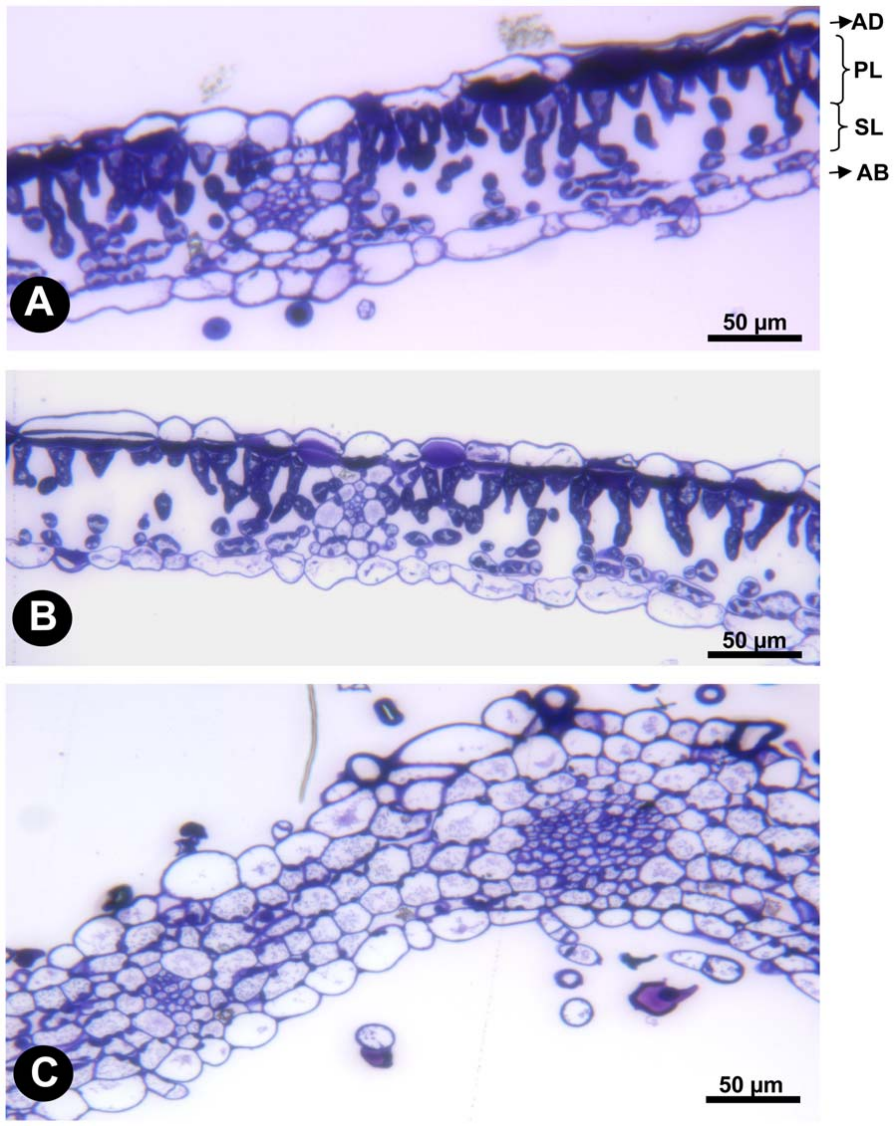
Cellular organization of leaf of wild-type and *FaKNOX1* RNAi plant. Cross sections through the (A) wild-type expanded leaf, (B) M1 *FaKNOX1* RNAi expanded leaf and (C) M2 *FaKNOX1* RNAi expanded leaf to show the variation in the arrangement of different cell layers. AD, adaxial epidermis: PL, palisade layer; SL, spongy layer and AB, abaxial epidermis. Bars = 50 µm.

The macroscopic variation in leaf morphology suggested that fine cellular differences might present as well. To assess the *FaKNOX1* RNAi effect on cellular organization, leaf cross sections from M1 and M2 plants were examined. Generally the wild-type leaf consists of adaxial and abaxial epidermis and middle mesophyll layer (Figure 8A). The mesophyll layer is subdivided into palisade and spongy layers. The palisade layer has tightly packed and elongated cells whereas the cells of the spongy layer are more rounded and loosely distributed. As mentioned earlier, the M1 RNAi plant shows three types of leaves. For anatomical studies only one type of leaf morphology was selected. In this class, the leaflets showed the least severe defect (Figure 7A) and except for small size appeared similar to wild-type. Leaves from M1 plants showed cellular structure similar to wild-type (Figure 8B). Examination of the M2 plants showed that differentiation of mesophyll layer was not apparent, and the space was occupied by big, round and tightly packed cells (Figure 8C).

**Figure 9:**
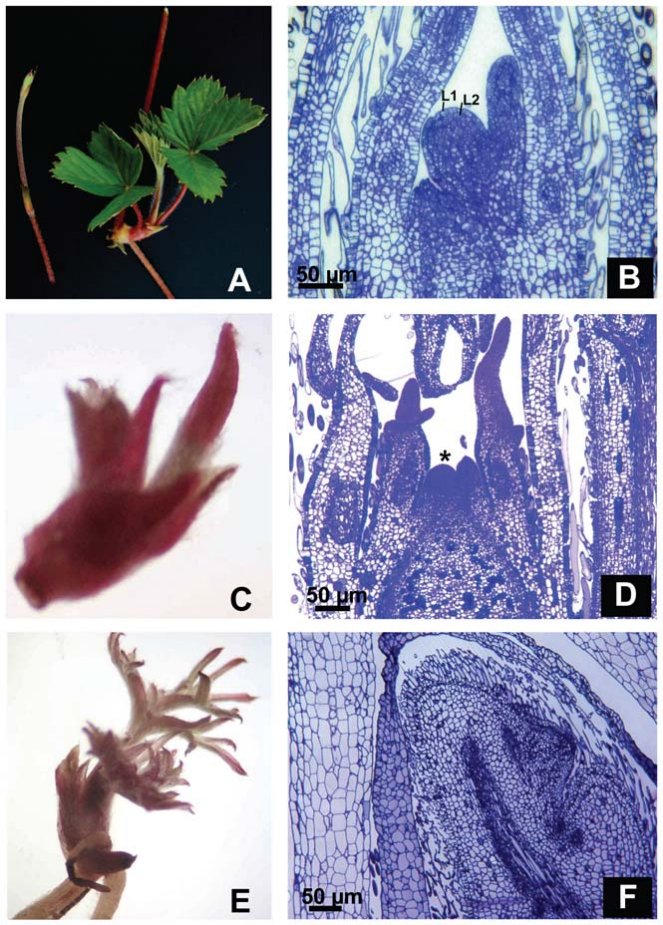
Structural organization of the shoot apical meristem of wild-type and *FaKNOX1* RNAi plants. (A) Runner tip and emerging plantlet from the stolon of wild-type strawberry plants, (C) runner bud and (E) young developing plantlet from *FaKNOX1* RNAi plants showing pale colored, narrow and elongated leaves. Transverse section through the shoot apical meristem of wild-type (B) and different stages from developing runner tip of *FaKNOX1* RNAi plants (D and F). Asterisk indicates SAM. Bars = 50 µm.

Expression analyses showed elevated transcript levels in the runner tip, a structure supporting rapid differentiation of new leaf materials. To investigate whether *FaKNOX1* RNAi affected the development and/or topology of the shoot apical meristem, longitudinal sections of different developmental stages of runner tip were analyzed. It was observed that the SAM of wild-type *F. vesca* is dome-shaped and composed of several layers of cells with outermost layer tunica and innermost corpus. Developing leaf primordia were observed at the flanks of the SAM (Figure 9A, 9B). The SAM of *FaKNOX1* RNAi young runner tips also showed a dome-like structure with developing leaves (Figure 9C, 9D). As the runner tip developed the SAM grew in size and small bulges corresponding to formation of leaves were observed (Fig. 9E, 9F). The cellular structure of the outer layer showed a high degree of disorganization. In place of round and compactly arranged cells, disorganized elongated cells were observed. Some cells also show hair-like structures. These data indicate that suppression of *FaKNOX1* expression causes deviations from normal SAM development in strawberry.

### Overexpression of *FaKNOX1* in *Arabidopsis* and Strawberry

**Figure 10:**
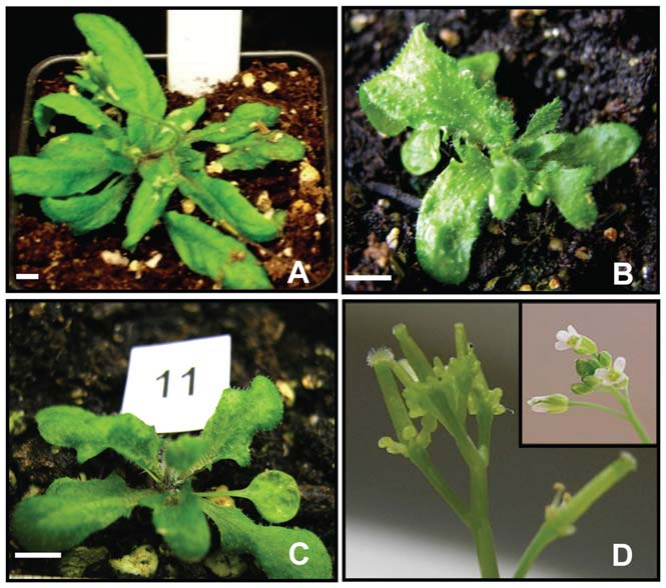
*Arabidopsis* plants overexpressing *FaKNOX1*. Phenotype of wild-type *Arabidopsis* plants (A) and plants overexpressing *FaKNOX1* (B and C). Overexpression lines present deeply lobed leaves, asymmetrical adaxial/abaxial growth, and a severe dwarf phenotype. (d) Flowers of *FaKNOX1* overexpression lines show early abscission of petals and stamens that fail to develop (inset = wild-type flowers). Bars = 5 mm.

**Figure 11:**
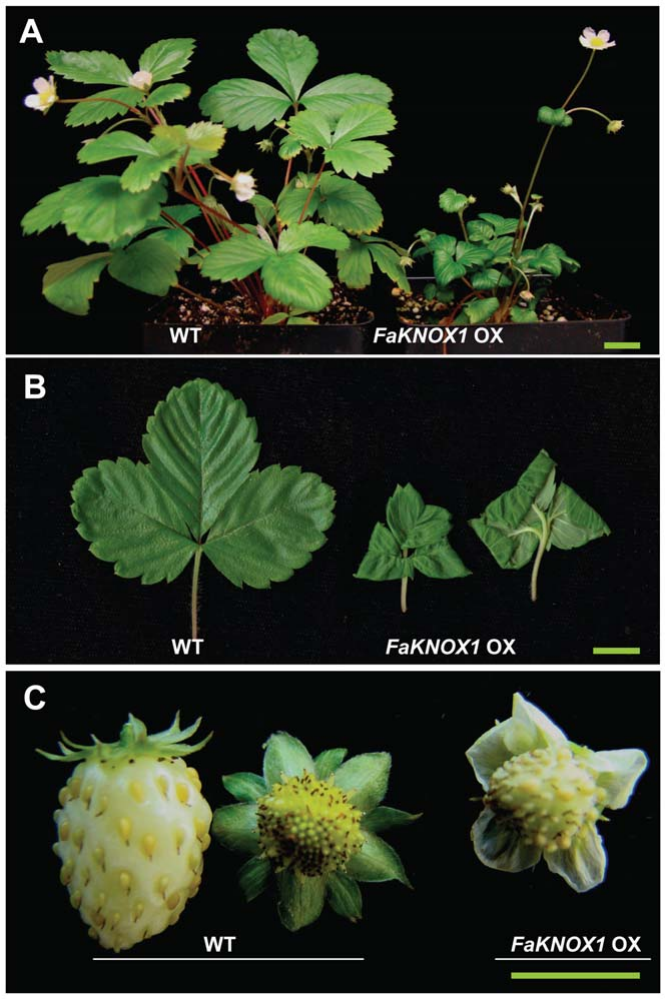
Strawberry plants overexpressing *FaKNOX1*. (A) Comparison of phenotypes of wild-type and *FaKNOX1* overexpression transgenic plants. (B) Phenotype of wild-type leaf with leaf forms obtained from *FaKNOX1* overexpression plant. (C) Comparison of phenotype of wild-type fruits with *FaKNOX1* overexpressing fruit. Note that the petals of wild-type fruit have abscised prior to fruit expansion whereas the *FaKNOX1* overexpression plants produce fruits where petals fail to abscise. Bars = 1 cm.

To determine the effect of ectopic expression of *FaKNOX1* on the leaf growth and development, the full-length *FaKNOX1* cDNA was expressed from a CaMV 35S promoter. The construct was first tested in *Arabidopsis*. Over twenty T0 independent *Arabidopsis* lines were selected as described in Materials and Methods. Ectopic overexpression of *FaKNOX1* in *Arabidopsis* led to dwarfed rosettes and low fertility, producing approximately 1% the seed of normal plants. The leaves were highly lobed (Figure 10B, 10C) and the flowers presented short, thickened stamens (Figure 10D) that produced little pollen. The petals appeared normal but easily abscised following minimal contact. At least twenty T0 independent overexpression lines were also analyzed in strawberry. RNA-gel-blots were performed to verify overexpression in these lines (Figure S3). Overexpression of *FaKNOX1* resulted in severely dwarfed plants with highly wrinkled and curled leaves, as the adaxial side was larger than the abaxial (Figure 11A, 11B). These plants produced prolific flowers on long pedicles but rarely generated fruits. Many of these plants generated seeds and T1 generation plants maintained the phenotype. Flowers appeared relatively normal with some flowers producing slight serrations on the petals. Some flowers exhibited a delay in, or complete lack of petal abscission (Figure 11C).

## Discussion

The understanding of the molecular control of leaf morphology has been greatly accelerated by the definition of genes that contribute to the formation of simple or compound leaves. In this report we present foundational analyses on a Class I *KNOX* gene that plays a role in regulation of plant stature and leaf morphology in strawberry. The strawberry system is a unique backdrop for this work. First, strawberry is a member of the Rosaceae family, a group of valuable fruit, nut and ornamental plants that rely heavily on organ shape and size to present marketable plant products. Second, strawberry plants have a distinctive leaf developmental pattern. Emerging strawberry leaves start out as simple leaves for the first weeks of plant development, but as the crown matures, the simple leaf form becomes increasingly complex, transitioning to the familiar three-lobed leaf. The present report provides groundwork to explore this process.

The *FaKNOX1* sequence was obtained from a survey of *F. × ananassa* flower cDNA sequences that did not match any GenBank database sequences with high similarity at the time of identification. In an effort to understand the function of this novel sequence, loss- and gain-of-function transgenic plants were generated and yielded conspicuous phenotypes. Further investigation indicated that *Fa*KNOX1 belongs to the Class I KNOTTED-LIKE HOMEODOMAIN group of proteins. *Fa*KNOX1 contains the basic domain organization of proteins in this family, including a MEINOX domain, GSE domain, ELK domain and homeodomain with PYP three amino acid loops (Figure 2A). Genetic linkage analysis places *FaKNOX1* on diploid (FV×FN) linkage group VI, bin 14.

The *FaKNOX1* transcript was present to a detectable level in all the organs examined, including runner tip (containing vegetative shoot apex), leaf, flower, fruit and root (Figure 3A, 3B) of *F. vesca* plant. The transcript was more abundant in flowers and fruits, in contrast to earlier studies that show that expression of most of the Class I genes is generally restricted to the SAM and floral meristem [1]. The observation that these transcripts are present at substantially higher levels in the strawberry mature fruit and flower supports the hypothesis that *FaKNOX1* is necessary for normal patterning in these tissues. This hypothesis is supported by analysis of transgenic plants, where defects in flower form, petal abscission, fruit set, and fertility were observed (Figures 4, 10 and 11b). Low levels of the transcript were detected in single- and multiple-leafleted leaves of *F. vesca*, indicating that FaKNOX1 is not solely responsible for the variations in leaf morphology (Figure 4).

However, RNAi suppression of *FaKNOX1* resulted in dwarfed plants with conspicuous alterations in leaf morphology. Short stature is also observed in a loss-of-function mutations of *OSH15* (rice) and *KNAT1* (*Arabidopsis*) [36], [37]. Deeply serrated, parsley-like leaves with abnormally long petiolules (Figure 7A) were common among all RNAi lines. The leaf phenotypic effects generated by suppression of *FaKNOX1* indicate that *FaKNOX1* does not function identically to other Class I *KNOX* genes in other species. This difference may be attributed to the fact that *FaKNOX1* is grouped with a separate subclass of KNOX proteins, in this case the OSH6 Class Ib KNOX proteins (Figure 2B). The functional comparisons between the roles of *Fa*KNOX1 and other OSH6 Class Ib KNOX proteins indicate that while most of members in this class affect leaf development, they do so in different and in some cases contrasting ways. Ectopic expression of the monocot orthologs, *ZmLG3* and OSH6 resulted in blade-to sheath transformation as well as loss or displacement of the ligule and auricles [30], [38]. Overexpression of NTH1 in tobacco resulted in curved leaves with a normal height [39] whereas overexpression of *Fa*KNOX1 results in dwarf plants with wrinkled and curved leaves with asymmetrical growth between abaxial and adaxial layers (Figure 11B). Many reports indicate that down regulation of gibberellin biosynthesis leads to dwarf stature in KNOX overexpressing plants [19], [40]. Comparative studies in families like Papaveraceae, Gesneriacea, Brasicaceae and simple and complex leaf species of *Lepidium* also illustrate the contrasting patterns of Class I *KNOX* gene expression in the control of leaf morphology [2], [23], [41], [42], [43]. Thus the final leaf form obtained is dependent on Class I *KNOX* genes, but the roles and mechanisms differ between species. Here the loss-of-function effects on strawberry plantlets add another piece of information to this understanding.

Two distinct classes of phenotypic severity were observed among *FaKNOX1* RNAi lines (Figure 6), even though suppression was complete in all the tested lines (Figure S1). While difficult to reconcile this finding, it is possible to speculate that the differences observed arose from variable RNAi suppression in specific cell types or more robust compensatory changes from paralogs that were confined to specific tissues (Figure S2). The mesophyll layer of severely affected RNAi leaves does not have distinguishable palisade and spongy layers (Figure 8). The cells are tightly arranged and are much larger in size than in comparable wild-type leaf tissues. A similar disorganization of mesophyll layer was observed with ectopic overexpression of *POTH1* in potato and *KNAT1* and *KNAT2* in *Arabidopsis*. In contrast to *FaKNOX1* RNAi effects, the leaves from these overexpression plants feature small cells or cells with a greater nucleus/cytoplasmic volume ratio [18], [20], [40], [44]. In another study, Hay and Tsiantis (2006) demonstrated that *STM* RNAi lines in *C. hirsuta* showed increased cell expansion and decreased expression of cell cycle markers. Thus during early stages of leaf initiation KNOX transcription factors drive leaf development in various ways in different species by controlling the temporal action of cellular growth and differentiation pathways.

In the present study the RNAi analyses were performed with the caveat that *FaKNOX1* is a member of a gene family and RNAi suppression could affect other family members. Analysis of sequences present in EST collections, the recently sequenced diploid strawberry genome and contigs derived from high-throughput sequencing suggests that *FaKNOX1* bears only 86–96% identity at stretch of 80-30 nucleotides with four paralogous sequences [45]. Analyses via relative real-time quantitative PCR of available *KNOX* sequences provided minor evidence of collateral suppression of only *FvKNOX2* (Figure 2). The different classes of *FaKNOX1* RNAi phenotypes may also arise due to the slight suppression of *FvKNOX2* transcript.

Thus phenotypes observed across RNAi plants are likely due to direct suppression of *FaKNOX1* transcripts. However, we cannot rule out a partial effect of compensatory changes from paralogous genes. Overexpression of other Class I or undescribed *KNOX* genes in response to suppression of *FaKNOX1* could possibly underlie phenotypes observed, as these are reminiscent of overexpression phenotypes in other systems. While a formal possibility, we have no evidence to support this hypothesis. Such overexpression could be occurring in discrete cellular contexts or developmental stages that would have profound effects but would be difficult to detect.

*FaKNOX1* RNAi strawberry plants rarely flowered. Flowering was generally suppressed with an occasional pedicle developing on some plants. Like the leaves, the petals were invaginated compared to those from a wild-type plant (Figure 7B, 7C) and these plants failed to produce fruits. Overexpression of *FaKNOX1* in *Arabidopsis* led to flowers with normal floral organization that showed early abscission of petals (Figure 10). In strawberry a contrasting phenotype was observed, as overexpressing transgenic lines exhibited marked delay in petal abscission (Figure 11C). These findings indicate *FaKNOX1* may also have a role in the abscission of petals, and which is regulated in different ways among diverse species. In tomato, *KNOTTED TKN4* is expressed at a higher level in the abscission zone of pedicel compared to the non-abscission zone. Pretreatment with the ethylene action inhibitor 1-MCP completely inhibited pedicel abscission and led to reduction of expression of *TKN4* [46]. Severe inflorescence and floral defects were also observed in a loss-of-function mutant of maize *KNOTTED1* [47] and a gain-of-function mutant of soybean *GmKNT1* [48]. Overexpression of *OSH15* in tobacco generated malformed flowers with low fertility [49]. Thus along with leaf development KNOX plays a significant role in floral architecture and function, consistent with its relatively high level of expression in these tissues.

In *Arabidopsis* the *STM* gene is a central player in shoot meristem formation and maintenance [12], [16], [50], [51]. EMS mutants of *STM* fail to produce a SAM and gain-of-function mutants in tobacco produce ectopic shoots. Other subfamily members like *KNAT1/BP* and *KNAT6* can function redundantly with *STM* for the SAM maintenance [18], [29], [52]. However, the loss of *KNAT2* does not have an effect on the SAM maintenance and function [29]. Loss of *FaKNOX1* perturbed the organization of the SAM (Figure 9D, 9F). The cellular structure of the outermost tunica layer showed elongated cells with hair-like structures. Small bulges corresponding to the formation of lateral organs were observed. These disturbances in the early SAM development likely contributed to the eventual altered leaf phenotypes. Meristems from strawberry RNAi runner tips exhibited alterations in early leaf morphology, supporting this interpretation. These results indicate a role of *FaKNOX1* in maintenance and differentiation of the developing meristem that affects long-term leaf morphology and cellular organization.

Across plant species, expression of *KNOX* genes is a crucial factor in determination of simple or compound leaf morphology. Studies in simple-leaved species like *Arabidopsis*, tobacco, maize and rice indicate that at the early stage of leaf development *KNOX* expression is down-regulated [17], [32], [39], [53]. In contrast, the presence of *KNOX* expression in tomato leaves contributes to the compound leaf form [21], [22]. Recently, Muller et al. [54] has demonstrated that ectopic expression of *KNOX* results in a developmental shift from simple to compound leaf form in dandelion (*Taraxacum officinale Web*.). Similarly RNAi lines of the *STM* ortholog in *Cardamine hirsuta* show reduction in the number of leaf lobes whereas ectopic expression of KN1 leads to increased leaflets number [23], [24]. *FaKNOX1* RNAi plants consistently produce few or no single-lobed leaves during early development. Three-lobed shoots emerged directly from callus-based organogenesis in RNAi plants, contrasting against wild-type shoot induction that only produced single-bladed leaves (not shown). Low levels of *FaKNOX1* transcripts were always detected in all stages of leaf development, including in strawberry seedlings during trifoliate leaf morphogenesis from single-bladed leaf forms (Figure 4). Data obtained from *in situ* hybridization of the SAM and leaf primordium regions indicated the presence of *FaKNOX1* at early stages in developing leaves and in the apical dome (Figure 5). These observations, in conjunction with loss- and gain-of-function studies in transgenic plants, supports the interpretation that *Fa*KNOX1 participates in establishing normal cellular organization and leaf morphology, and that the roles are most pronounced during early leaf development. There are no data in this report that indicate a clear role for *Fa*KNOX1 in the developmental progression from single-leaflet to trifoliate leaves, as the transcript is detected at low levels in leaves from both stages (Figure 4E).

In conclusion, the *FaKNOX1* gene has clear roles in strawberry leaf development, cellular organization and eventual floral, fruit and leaf morphology. Additional genetic and genomic tools exist to further delineate the roles of *Fa*KNOX1 genes in control of strawberry form. The complete set of *KNOX* genes may be analyzed in the agile *F. vesca* transformation system. During his time at the Versailles botanical gardens Duschene noted the unusual features of a plant now recognized as *F. vesca monophylla*, a line of *F. vesca* that always produces single-leafleted leaves. This strawberry variant also may become an important resource for further study. This first report presents the foundation for further studies leveraging the unique attributes of the strawberry system.

## Materials and Methods

### Isolation of *FaKNOX1*

The full-length *FaKNOX1* transcript was isolated by random Sanger sequencing of cDNA clones from a *Fragaria × ananassa* flowering library. The genomic sequence of *FvKNOX1* was obtained from *F. vesca* Hawaii-4 by PCR amplification using primer pair 5′-CCCACTGGGACTTCATTCTTCT-3′and 5′-GGCCCAAATAATGGAAATTTAGAG-3′. The sequence of the genomic and cDNA clones are available in the Genbank as GU339211 (Gene) and GQ465832 (cDNA).

### Linkage Mapping of *FaKNOX1*

Linkage mapping was performed from DNA obtained from lyophilized *F. vesca* tissue. The Fv×Fn F2 generation of bin mapping plants [34] were used for these analyses. DNA was isolated using the DNeasy Plant Mini Kit (Qiagen, Valencia, CA) and the TissueLyser (Qiagen, Valencia, CA) procedure for lyophilized tissue. Gene specific primers 5′-TCCACCAGAGATTAAAAGCCTACT-3′ and 5′-TCAATGTTGTTCAAGAATGTGGTT-3′ were used for amplification of a polymorphic region of *FaKNOX1*.

PCR reactions were prepared with 20 ng genomic DNA template with AccuPrime *Taq* DNA Polymerase High Fidelity (Invitrogen, Carlsbad, CA), following the manufacturer's protocol. Reactions were amplified with an initial denaturation of 94°C for 2 min, followed by 35 cycles of 94°C for 30 s, annealing at 53°C for 30 s and elongation at 68°C for 40 s, followed by a final elongation at 68°C for 10 min.

Genotypes of F2 plants were assigned on the basis of sequences obtained from direct sequencing of PCR products. Sequencing templates (10 ng each) and primers (2 µL at 2 µM each) were provided for sequencing using BigDye Terminator Cycle Sequencing Kits (ABI) on the ABI3130 DNA Analyzer at the Hubbard Center for Genome Studies (University of New Hampshire, Durham).

The *FaKNOX1* gene was mapped on the basis of DNA sequence differences between mapping parents Fn601 and Fv815. When DNA chromatograms were visualized, clean unambiguous sequences were observed for plants 32, 42, 46, and 62, and these sequences exactly matched those of parent Fv815. Thus, these plants were homozygous for the Fv815 allele, and were coded “a”. Conversely, the chromatograms for plants 59 and 83 displayed superimposed peaks, yet through careful visual deconvolution it was possible to discern the presence of Fv815 and Fn601 alleles, both of which had been sequenced independently. Thus, plants 59 and 83 were heterozygous, and were coded “h”. The genotypes were compared with the FV × FN Bin Map code sheet provided by D. Sargent (East Malling Research, UK), allowing assignment of the gene to its linkage group and bin.

### RNA isolation, RT-PCR and real-time PCR

Total RNA was isolated from various tissues using a modification of a CTAB-based method [55]. Tissue-specific expression analysis was performed using RNA-gel-blot analysis and quantative real-time PCR. Results obtained from both analytical methods, performed with two independent biological replicates produced nearly identical results. RNA-gel-blot analysis was performed as described [56].

Real-time PCR was performed using primer pair 5′*FaKNOX1*-AATGGTGAAGGACCAAGTT-3′, 5′*FaKNOX1*-TTCAACCTGCTCATTATTGCCTA-3′ and internal control (Elongation factor 1 alpha; EF1a) 5′*EFL1A*-GCCCATGGTTGTTGAAAGTTTC-3′ and 5′*EFL1A*-GGCGCATGTCCCTCACA-3′. The CT (cycle threshold) values for all genes in different RNA samples were normalized to the CT value of an internal control gene, *EF1A*. The relative mRNA levels for each candidate gene in different tissue samples were calculated using the ΔΔCT method (StepOnePlus Real-Time PCR System, Applied Biosystems, USA). Error bars in the figures indicate standard error of the mean.

For two-step RT-PCR a standard reverse transcription protocol was performed using ImProm-II reverse transcriptase (Promega, USA). The primer pair used was 5′*FaKNOX1*-GTAGGTCGGCTAAGCTTCCCA-3′ and 5′*FaKNOX1*- GCTGCCTGAAGGTCACAGTTG -3′. PCR conditions were: 2 min at 94°C; 38 cycles (30 s at 94°C; 30 s at 55°C; 20 s at 72°C); followed by a 10 min final extension step at 72°C. *FaUBQ* (the most similar strawberry homolog to *Arabidopsis UBQ10*) served as the internal control.

### *In situ* hybridization

Shoot apices of 20-d-leaf and 45-d-leaf stage seedlings were dissected out and fixed in cold formalin acetic alcohol (FAA; 3.7% formaldehyde, 5% acetic acid and 50% ethanol) for 24 h, followed by dehydration through an ethanol series and embedded in Paraplast Plus paraffin. cDNA fragment specific to *FaKNOX1* was amplified using primer pair 5′-AACCCACTGGGACTTCATTC-3′ and 5′-GGTAGCCACTGTACTTATGCA-3′. Sense and antisense RNA probes of *FaKNOX1* were labeled using the DIG RNA labeling kit according to manufacturer's instructions (Roche Diagnostics GmbH, Mannheim, Germany). The probe was hydrolyzed using 0.2 M carbonate buffer at 60°C for 51 min. For detection of *FaKNOX1* transcripts the sections were pre-treated, hybridized, washed and developed according to Jackson [57]. The sections were viewed and photo documented with a Nikon Digital Sight DS-Fi1 camera on a Nikon AZ100 light microscope.

### RNAi and overexpression constructs

The full-length *FaKNOX1* cDNA was shuttled into pK7GWIWG2D (for RNAi) and pH7WG2D (for overexpression) binary vectors [58] using standard clonase reaction conditions (Invitrogen, Carlsbad, CA). For the RNAi construct the full cDNA sequence was used because a substantial portion of the transcript lacked similarity to known transcripts at the time of cloning. Both vectors contain a GFP cassette on a separate promoter for identification of transgenic callus. The correct orientation and insertion sizes were confirmed by restriction digestion and by sequencing of recombination borders. Constructs were introduced into *Agrobacterium* strain GV3101 by electroporation.

### Strawberry transformation for RNAi and overexpression transgenic plants

For transformation and regeneration, leaf sections and petioles from *in vitro* grown *F. vesca* plants (Hawaii-4 genotype) were co-cultivated with *Agrobacterium* harboring an RNAi or overexpression construct in media containing MS salts, Gamborg vitamins, 2% sucrose, 3 mg/L benzyladenine, 0.2 mg/L indole-3-butyric acid and 0.7% agar [35], [59]. After 2 d of co-cultivation, explants were washed and transferred to media containing 3 mg/L benzyladenine, 0.2 mg/L indole-3-butyric acid, 5 mg/L kanamycin or 5 mg/L hygromycin) and 250 mg/ml cefotaxime. Explants were subcultured every 14 days until shoots appeared, typically 60–90 d. Healthy shoots were transferred to rooting media (0.01 mg/L of IBA, 2% glucose, 0.5× MS salts, 0.7% phytoagar, pH 5.8). The GFP-positive rooted plants were transferred to soil, acclimated to ambient temperature and humidity, and then moved to greenhouse conditions.

More than 20 RNAi lines and overexpression lines were generated and were clearly discernable by the presence of GFP and alterations in leaf morphology. The presence of many independent lines and agreement between GFP and the leaf phenotype indicates that the phenotypes observed are related to successful expression of the target sequence.

### *Arabidopsis* transformation for overexpression transgenic plants

The overexpression construct was transferred to *Arabidopsis* using the floral dip method [60]. Transgenic seedlings were selected on MS medium using hygromycin selection, and resistant/GFP-positive lines were selected. Further these lines were selfed for T1 and T2 generation.

### Light Microscopy

Runner tips and leaf sections of strawberry were fixed in 3.0% gluteraldehyde in 0.1 M sodium cacodylate pH 7.4 overnight at 4°C. Following fixation, materials were rinsed with buffer and fixed again with 2% osmium tetroxide in 0.1 M sodium cacodylate. Samples were dehydrated through a graded ethanol series. The tissue was then transferred to acetone and embedded in spur resin and polymerized at 70°C. Semi thin sections of 0.5 micron were prepared using ultra microtome (Power Tome X, Boeckeler). The samples were stained with toluidine blue-O and examined with Olympus-BH2-RFCA microscope.

### Phylogenetic analysis and accession numbers

The neighbor-joining method (Saitou and Nei, 1987) was used for phylogenic clustering which were conducted by MEGA4 [61]. Bootstrap values were based on 1000 replicates [62]. Accession numbers used in the relationship analysis are provided as a Table S1.

## Supporting Information

Figure S1 RT-PCR analysis for detection of full-length *FaKNOX1* transcript from four independent lines of *FaKNOX1* RNAi transgenic plants. Flower and runner tip mRNA were prepared as tissue samples as *FaKNOX1* is expressed in high abundance in these tissue. Based on availability of samples some lines were tested with flower and some with runner tip sample. *ACTIN* was used as an internal control.
(TIF)

Figure S2 Real-time PCR analysis for quantitation of transcript of different members of *KNOX* family members in WT and *FaKNOX1* RNAi lines. Runner tip was used as tissue samples as *KNOX* is expressed in high abundance in this tissue. Data from four *FaKNOX1* RNAi lines are presented here, three lines from M1 class and one from M2 class.
(TIF)

Figure S3 RNA-gel-blot analysis for quantitation of *FaKNOX1* transcript from four independent lines of *FaKNOX1* OX transgenic plants. RNA was prepared from mature leaves for overexpression lines.
(TIF)

Figure S4 A relative real-time PCR analysis to show tissue-specific expression of *FvKNOX2 - FvKNOX5* transcripts. The flower sample was used as reference tissue. Error bars represent standard error of the mean derived from three replicates.
(TIF)

Table S1 Accession numbers and their corresponding genes used for phylogenetic clustering.
(DOC)
