# Supplementary figures and images for: A Strawberry KNOX Gene Regulates Leaf, Flower and Meristem Architecture
Source: PLoS One. 2011 Sep 20;6(9):e24752. doi: 10.1371/journal.pone.0024752 (PMC3176782; doi:10.1371/journal.pone.0024752)

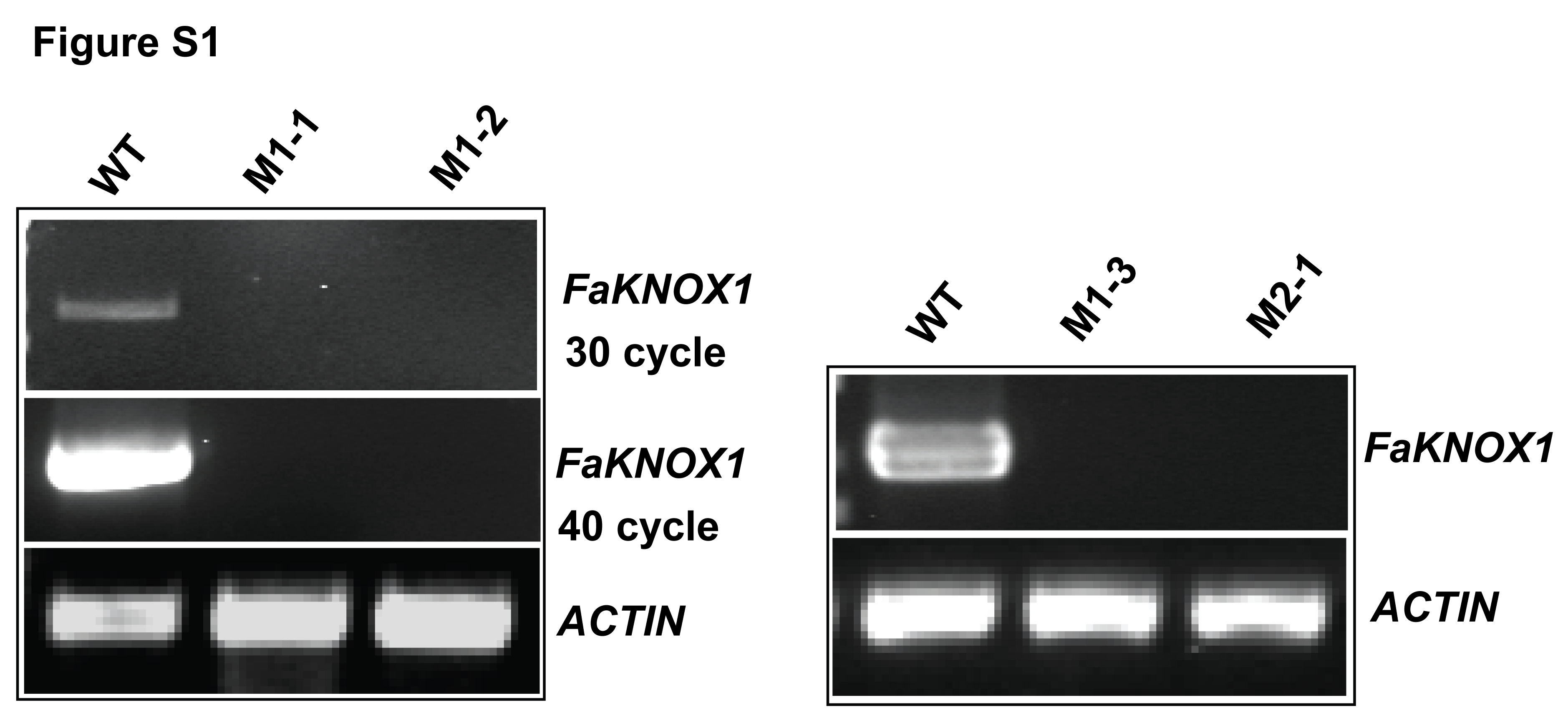

Supplement: Figure S1 — RT-PCR analysis for detection of full-length FaKNOX1 transcript from four independent lines of FaKNOX1 RNAi transgenic plants. Flower and runner tip mRNA were prepared as tissue samples as FaKNOX1 is expressed in high abundance in these tissue. Based on availability of samples some lines were tested with flower and some with runner tip sample. ACTIN was used as an internal control. (TIF) [file pone.0024752.s001.tif]

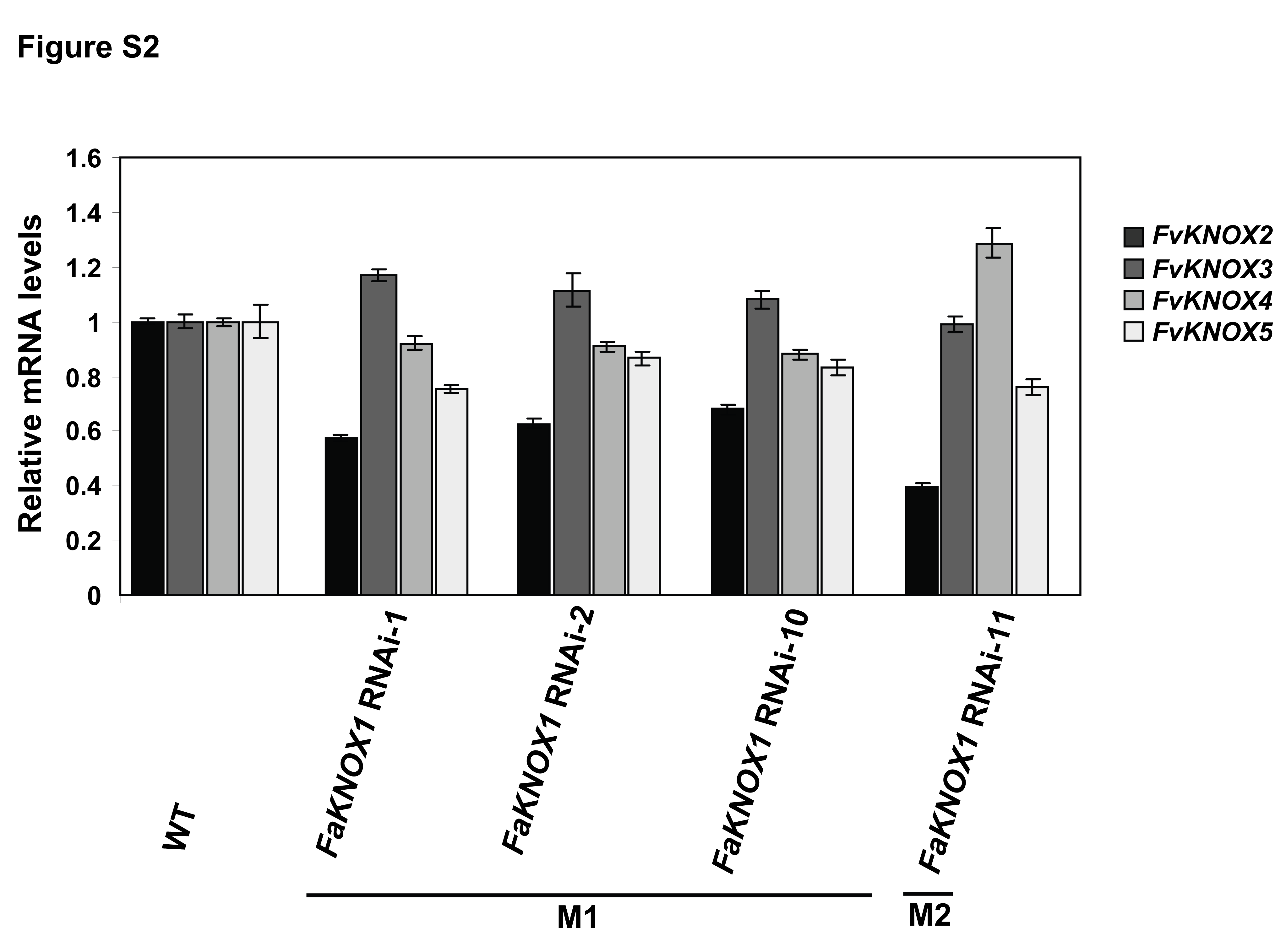

Supplement: Figure S2 — Real-time PCR analysis for quantitation of transcript of different members of KNOX family members in WT and FaKNOX1 RNAi lines. Runner tip was used as tissue samples as KNOX is expressed in high abundance in this tissue. Data from four FaKNOX1 RNAi lines are presented here, three lines from M1 class and one from M2 class. (TIF) [file pone.0024752.s002.tif]

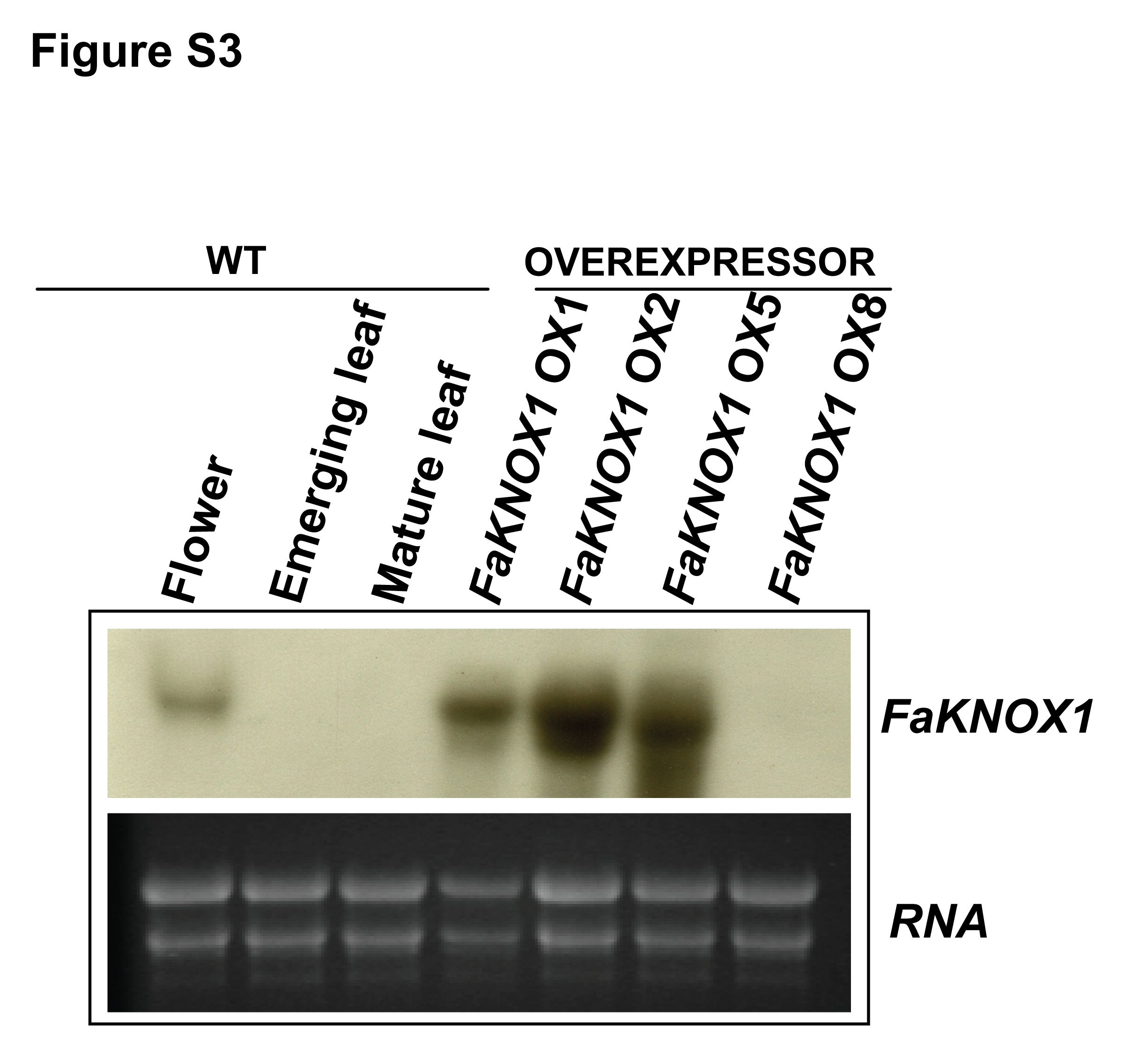

Supplement: Figure S3 — RNA-gel-blot analysis for quantitation of FaKNOX1 transcript from four independent lines of FaKNOX1 OX transgenic plants. RNA was prepared from mature leaves for overexpression lines. (TIF) [file pone.0024752.s003.tif]

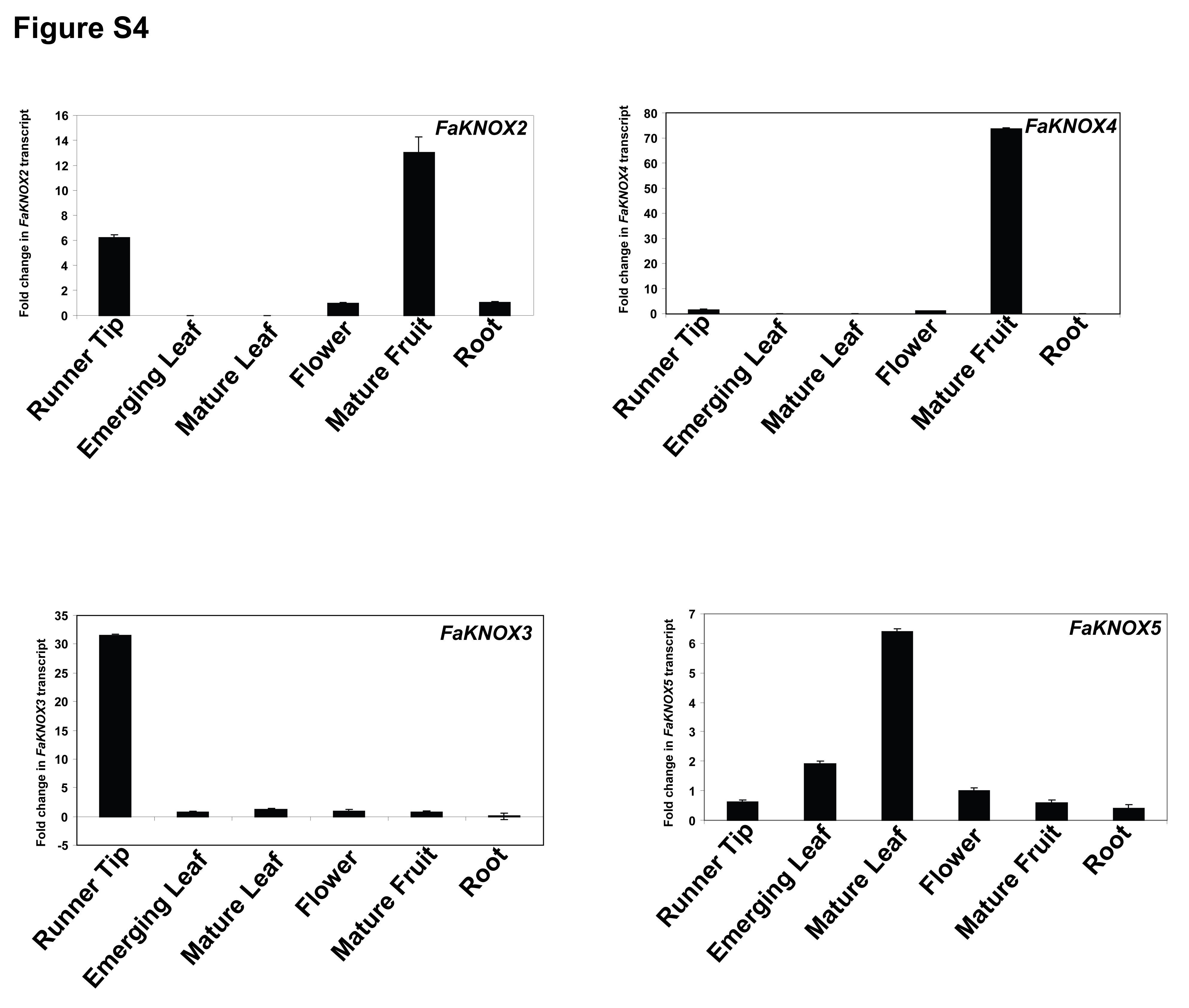

Supplement: Figure S4 — A relative real-time PCR analysis to show tissue-specific expression of FvKNOX2 - FvKNOX5 transcripts. The flower sample was used as reference tissue. Error bars represent standard error of the mean derived from three replicates. (TIF) [file pone.0024752.s004.tif]
