# Supplementary material for: A Strawberry KNOX Gene Regulates Leaf, Flower and Meristem Architecture
Source: PLoS One. 2011 Sep 20;6(9):e24752. doi: 10.1371/journal.pone.0024752 (PMC3176782; doi:10.1371/journal.pone.0024752)
Supplement: Table S1 — Accession numbers and their corresponding genes used for phylogenetic clustering. (DOC) [file pone.0024752.s005.doc]

**Table S1.**

Species names Gene names Accession no

*Arabidopsis thaliana* STM NP_176426

KNAT1 NP_192555

KNAT2 NP_177208

KNAT5 NP_194932

KNAT6 NP_850951

KNAT7 NP_564805

*Lycopersicon esculentum* TKN1 AAC49251

TKN2 AAD00251

TKN3 AAD00252

TKN4 AAP47027

*Nicotiana tabacum* NTH1 BAA76750

NTH9 BAA76903

NTH15 BAA25546

NTH20 BAA76904

NTH22 BAA76905

*Malus x domestica* KNAP1 O04135

KNAP2 O04135

*Prunus persica* KNOPE1 ABC84491

KNOPE2 ABO28750

KNOPE3 ABH03531

PpSTM2 ADC35600

*Fragaria vesca* FvKNOX2 HQ413775

FvKNOX3 HQ413776

FvKNOX4 HQ413777

FvKNOX5 HQ413778

*Oryza sativa* OSH1 JQ2379

OSH3 BAB68310

OSH6 BAA79224

OSH10 Q75LX7

OSH15 BAA31688

OSH43 BAA79225

OSH43A AAP68879

OSH71 A2Y007 KNOS5_ORYSJ Q75LX9 OSH45 BAA08552

HOS58 Q0E3C3

OSH45 Q0J6N4

HOS59 Q94LW4

*Zea mays*  ZmKN1 CAA43605

ZmLG3 NP_001105508

ZmLG4A AAP31409

ZmLG4B AAP31410

ZmRS1 NP_001105331

ZmGNARLY1 AAP76320

*Pinus Taeda* PtKN1 AAV54618

PtKN2 AAV54619

PtKN3 AAV54620

*Ceratopteris richardii* CRKNOX1 BAB18582

CRKNOX2 BAB18584

CRKNOX3 BAB18585
